# Nuclear transplantation between allogeneic cells through topological reconnection of plasma membrane in a microfluidic system

**DOI:** 10.1063/1.5098829

**Published:** 2019-06-10

**Authors:** Masahiro Okanojo, Kennedy O. Okeyo, Hiroko Hanzawa, Osamu Kurosawa, Hidehiro Oana, Shizu Takeda, Masao Washizu

**Affiliations:** 1 Center for Exploratory Research, Research and Development Group, Hitachi, Ltd., Hatoyama, Saitama 350-0395, Japan; 2 School of Engineering, The University of Tokyo, Hongo 7-3-1, Bunkyo-ku, Tokyo 113-8656, Japan; 3 Department of Biosystems Science, Institute for Frontier Life and Medical Sciences, Kyoto University, Yoshida-honmachi, Sakyo-ku, Kyoto 606-8501, Japan; 4 Department of Mechanical Engineering, School of Engineering, The University of Tokyo, Hongo 7-3-1, Bunkyo-ku, Tokyo 113-8656, Japan; 5 Department of Bioengineering, School of Engineering, The University of Tokyo, Hongo 7-3-1, Bunkyo-ku, Tokyo 113-8656, Japan

## Abstract

Previous studies have demonstrated that somatic cells fused with pluripotent stem cells can be reprogrammed on the basis of reprogramming factors acquired from the latter. However, fusion-reprogrammed cells are deemed unsuitable for therapeutic applications mainly because conventional fusion techniques often yield tetraploid fusants that contain exogenous genes acquired from the fusion partners. Here, we present a novel cell–cell topological reconnection technique and demonstrate its application to nuclear transplantation between a somatic cell and a stem cell without nuclei mixing. As a proof of concept, a microfluidic fusion chip embodied with a microslit (4 *μ*m in width) to prevent nuclei mixing was developed and used to perform one-to-one electrofusion of a target somatic cell (Jurkat cell) with an induced pluripotent stem (iPS) cell. To extract its cytoplasm, the target cell was first topologically connected to a sacrificial iPS cell by electrofusion via a microslit, followed by shear flow removal of the latter to obtain a cytoplasm-depleted nucleus of the target cell. Then, to replace the lost cytoplasm, topological reconnection to a second iPS cell was performed similarly by electrofusion, followed by shear flow separation of the target cell to enable it acquire most of the iPS cytoplasm, but without nuclei mixing. Microscopic observation of target cells harvested and cultured *post hoc* in a microwell confirmed that they manifested cell division. Taken together, these results demonstrate the potential application of the cell–cell topological reconnection technique to somatic cell nuclear transplantation for the generation of autologous pluripotent stem cells.

## I. INTRODUCTION

Previous studies have demonstrated that fusion of embryonic stem cells (ES cells) with somatic cells can initiate reprogramming of somatic cells to pluripotency by acquiring reprogramming factors from the stem cells.^1,2^ Consequently, cell fusion-based reprogramming is increasingly being applied toward understanding epigenetic modifications during the initiation of reprogramming or dedifferentiation.^3,4^ Conventionally, cell fusion has been achieved using viruses,^5^ polyethylene glycol,^6^ and various electrical approaches (i.e., electrofusion).^7,8^ However, these standard cell fusion techniques result in the random fusion of two or more cells that are in contact, leading to the formation of tetraploid or even higher degree polyploid fusants. Such cells are less attractive for therapeutic applications because of their tetraploidy and the presence of exogenous genes from stem cells.^9,10^ Moreover, the efficiency of conventional electrofusion depends heavily upon the relative sizes (diameter) of the cells involved.^7^ For example, high electric field strength is necessary to induce sufficient membrane potential in small cells, but this can destroy larger cells, resulting in low fusion efficiency especially when the difference in cell diameter is large.

To overcome these limitations, our group has developed a technique of one-to-one electrofusion via microslits or microorifices that utilizes electric field constriction to achieve fusion at relatively low voltage.^11–14^ Since the size of the microorifice used is 3–4 *μ*m, which is smaller than a nucleus (∼7 *μ*m), the nuclei of the fused cells do not mix but remain separated at opposite sides of the orifice.^15–17^ Only cytoplasmic contents of the two fused cells mix by diffusion, and this can be accelerated by applying a pressure difference across the orifice. Thus, with this fusion technique, the formation of tetraploidy or genetic contamination can be prevented. Moreover, unlike conventional electrofusion techniques, the fusion efficiency of the fusion via microslit technique is not affected by cell-size differences.^18^ One-to-one electrofusion, which essentially involves application of a potential to cause a reversible breakdown of the cell membrane, is attractive since it enables cell fusion easily and without chemical or genetic contaminations. In fact, in our previous study, we demonstrated the possibility of reprogramming mouse embryonic fibroblasts (MEFs) by one-to-one electrofusion with mouse ES cells.^19^ Cell division and OCT4-GFP (green fluorescent protein) expression by MEF was confirmed within 25 h of fusion.^19^ However, in this case, the nuclei of the hybrids/fusants could slip through the microslit and form heterokaryons during the M-phase, when the nuclear membrane breaks down. Thus, the cells monitored for reprogramming in this study were still tetraploid.^15,19^ To prevent the formation of tetraploidy, one strategy would be to separate and remove completely one of the nuclei soon after one-to-one electrofusion, but currently no technique exists for accomplishing this task in a microfluidic system.

**FIG. 1.**
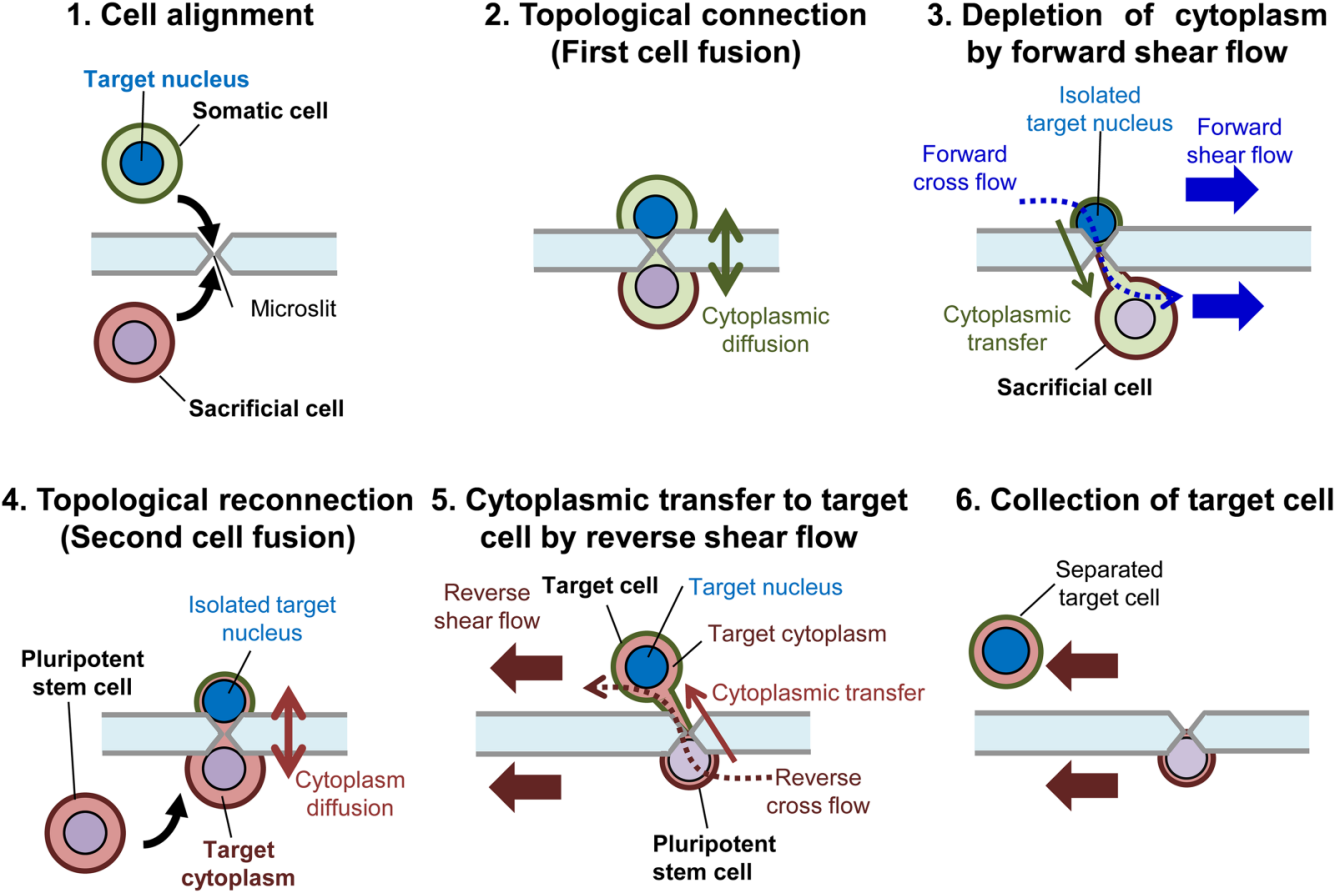
Concept of cell–cell topological reconnection method. Cell–cell topological reconnection combines the key techniques of one-to-one electrofusion and shear flow separation to achieve cytoplasmic transfer from a pluripotent stem cell to a somatic cell without nuclear gene mixing between the two-cell species. (1) Cell alignment of a somatic cell with its target nucleus and a sacrificial cell (e.g., a pluripotent stem cell) on a microslit prior to cell fusion. (2) Topological connection (first cell fusion) of the aligned cells to achieve passive cytoplasmic exchange by diffusion. (3) Isolation of target nucleus and extraction of somatic cytoplasm by application of a forward shear flow to deprive the somatic cell of its cytoplasm. (4) Topological reconnection (second cell fusion) is initiated between a pluripotent stem cell as its target cytoplasm donor and the cytoplasm-depleted somatic cell to achieve passive cytoplasmic exchange by diffusion. (5) Cytoplasmic transfer from a pluripotent stem cell into the target cell is further enhanced by application of a reverse shear flow. (6) Collection of the target cell, which now has its original target nucleus and target cytoplasm acquired from a pluripotent stem cell. Note that the nucleus of the pluripotent stem cell is not contained.

**FIG. 2.**
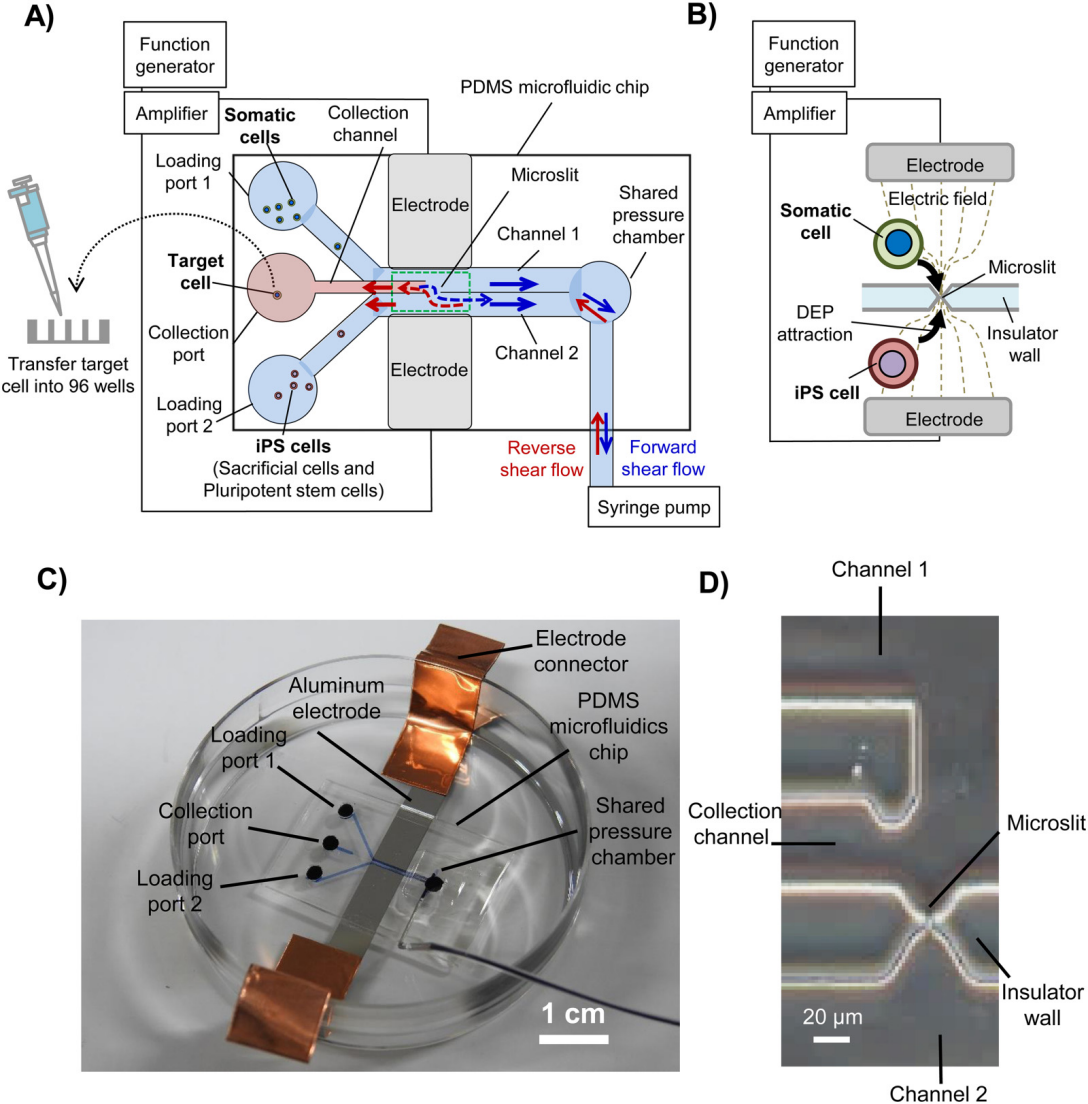
Device configuration for achieving cell–cell topological reconnection. (a) Schematic illustration of the device used in this study. The device consists of a PDMS microfluidic chip and two electrodes connected to a function generator via an amplifier. The PDMS chip consists of two loading ports for introducing the different cell species, which are contiguous with two main flow channels separated by an insulating PDMS wall where a microslit is located. A shared pressure chamber and a connecting syringe pump were used to control flow during the operations. Forward shear flow was generated by aspiration from the pump (solid blue arrows). Simultaneously, a forward cross flow (dotted blue arrow) passing thorough the microslit from the main flow channel 1 toward the main channel 2 was generated. Reverse shear flow was generated by discharging from the pump (solid red arrows). Contrary to the forward cross flow, reverse cross flow (dotted red arrow) passed thorough the microslit from the main flow channel 2 toward the main channel 1. A collection channel connecting to a collection port was used to harvest target cells, which was ultimately transferred manually from the collection port to a 96-well plate for culture and time-lapse monitoring. (b) Cell alignment by DEP at a microslit for cell fusion. (c) Actual image of the device. (d) Actual image of the portion of the chip containing a microslit and a collection channel. One-to-one cell fusion of the cell pair following pulse voltage application was confirmed by the spreading of a cell-permeant fluorescent dye used to stain the cells.

The primary objective of this study was to develop a novel technique for achieving transplantation of a somatic cell nucleus into the cytoplasm of a pluripotent stem cell without nuclei mixing. Nicknamed the “cell–cell topological reconnection technique” (Fig. 1), it combines one-to-one electrofusion with microfluidic operations to achieve cytoplasmic exchange between single cells on a single chip [Fig. 2(a)]. The name stems from the fact that cells, which are individually membrane-bound entities, can be considered as topoisomers that cannot be topologically transformed unless the cell membrane is first broken, as occurs during fusion.

As a proof of concept study, we applied our novel technique to transplant the cytoplasm of an iPS cell into the nucleus of a Jurkat cell (model somatic cell) by one-to-one electrofusion without nuclei mixing. Although not fully explored in this study, it is expected that pluripotency inducing factors contained in the iPS cytoplasm may trigger reprogramming of the somatic cell nucleus, enabling the generation of autologous pluripotent stem cells without tetraploidy formation and nuclei mixing in the future. Thus, with future improvements, the cell–cell topological reconnection technique presented in this study will enable the generation of comparatively safe iPS cells for possible applications to regenerative medicine.

### A. Concept of cell–cell topological reconnection technique

Essentially, the cell–cell topological reconnection technique integrates key techniques of one-to-one electrofusion and shear flow separation to generate a target cell consisting of a nucleus from a target somatic cell and a cytoplasm from a pluripotent stem cell (Fig. 1). In other words, the technique incorporates electrofusion via microslits and *post hoc* cell–cell separation to achieve nuclear transplantation between two single cells without nuclei mixing in a microfluidic system.

Briefly, in the first step, a somatic cell (target nucleus) and a sacrificial cell are fused (topological connection) one-to-one via a microslit to prevent nuclei mixing, and shear flow is applied to pull out the sacrificial cell, resulting in the withdrawal of the cytoplasm from the somatic cell to isolate its nucleus. In the second step, the retained somatic cell (with little cytoplasm) is again fused with a pluripotent stem cell in a topological reconnection manner, resulting in the acquisition of the stem cell cytoplasm (target cytoplasm). Finally, the target cell with the target nucleus and the target cytoplasm is similarly separated and collected by shear flow.

Figure 1 illustrates core procedures for cytoplasm withdrawal and transfer by shear flow manipulation during cell–cell topological reconnection. The major steps are as outlined below.

(1) Cell alignment [Fig. 1(1)]: A target somatic cell (with a target nucleus) and a sacrificial cell (e.g., a pluripotent stem cell) are loaded into separate channels of a microfluidic device and aligned to form a pair at a microslit by dielectrophoresis (DEP) [Fig. 2(b)].
(2) Topological connection [Fig. 1(2)]: To initiate topological connection, the pair is fused by pulsation, resulting in the cross-diffusion of the cytoplasmic contents between the fused cells even as their nuclei are kept apart by the microslit.
(3) Isolation of target nucleus after the first fusion [Fig. 1(3)]: A forward shear flow is applied to pull away the sacrificial cell from the target somatic cell. As a result, separation of the fused cell pair is achieved, with the target somatic cell losing most of its cytoplasm to the sacrificial cell to yield a target nucleus.
(4) Topological reconnection [Fig. 1(4)]: To initiate topological reconnection, a second pluripotent stem cell is introduced and fused with the cytoplasm-depleted somatic cell via the microslit. In this process, the cytoplasm-depleted target cell nuclear acquires the cytoplasm of the pluripotent stem cell (target cytoplasm), but their nuclei are kept separated by the microslit. The success of fusion is monitored by the diffusion of the dye between the cell pair.
(5) Cytoplasmic transfer after the second fusion [Fig. 1(5)]: A reverse shear flow is applied to pull away the target cell (with target nucleus and target cytoplasm) from the pluripotent stem cell and, in the process, the cytoplasm-depleted target cell nucleus acquires the cytoplasm of iPS cell.
(6) Collection of target cell [Fig. 1(6)]: A contiguous-reverse shear flow is applied to separate the target cell from the pluripotent stem cell and, in turn, harvest the target cell to a collection channel. Finally, the harvested target cell is transferred from the collection port to a 96-well plate for *post hoc* culture and microscopic observation.

**FIG. 3.**
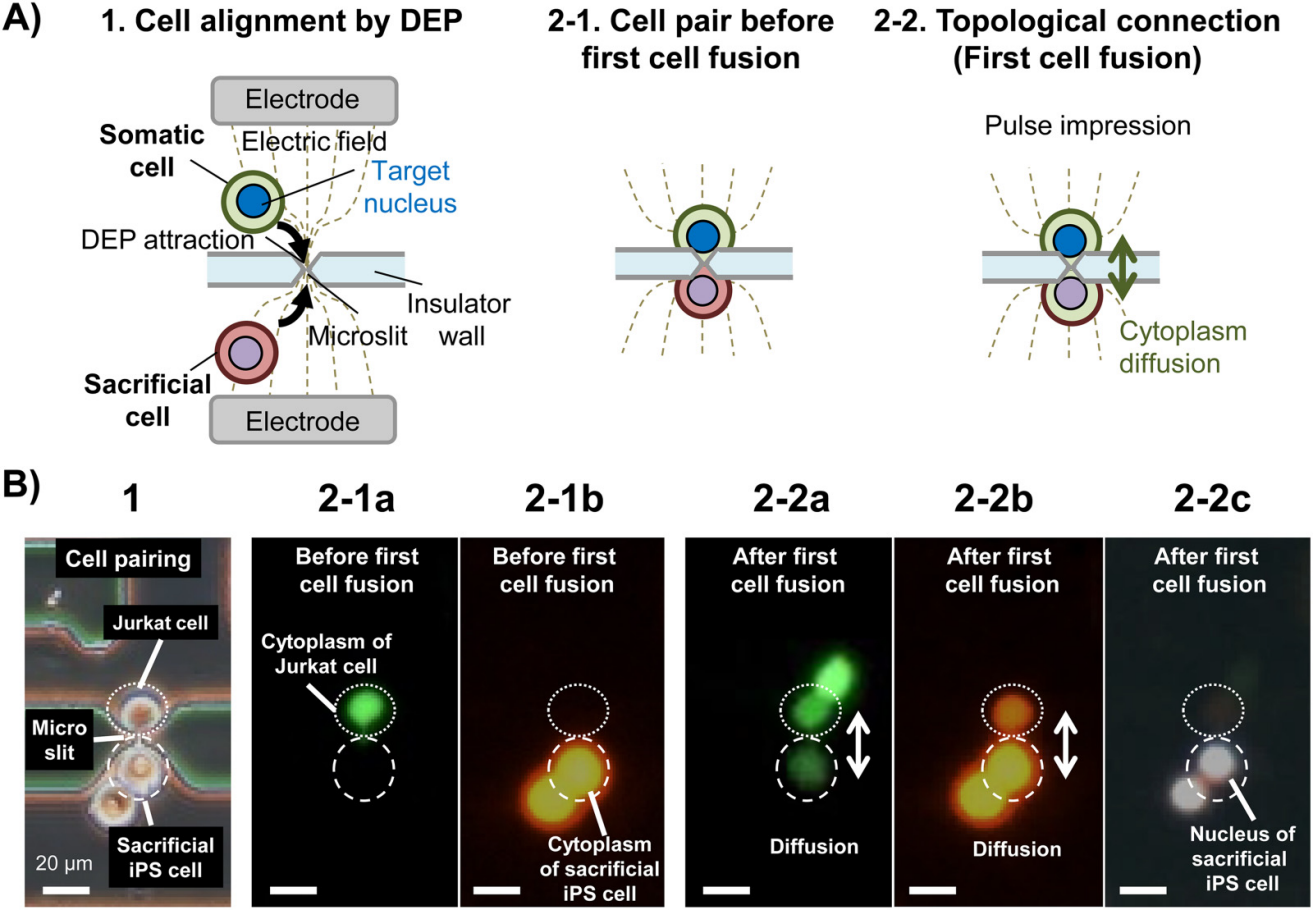
Results of topological connection during the first electrofusion via a microslit. (a) Illustration of events involved in the topological connection during the first cell fusion. Cell alignment by DEP at a microslit (1). Aligned cell pair before first cell fusion (2-1). Illustration of cytoplasm mixing after topological connection (the first cell fusion) (2-2). (b) Actual images of topological connection (first cell fusion) (video S1 in the supplementary material). An alignment pair of a Jurkat cell (model somatic cell) and a sacrificial iPS cell (first iPS cell) connected through a microslit (1). A fluorescence emitted by a fluorescently labeled Jurkat cytoplasm (stained with Calcein AM) (2-1a). Fluorescently labeled cytoplasm of sacrificial iPS cell (stained with Calcein Red-Orange) before first cell fusion (2-1b). Cytoplasmic mixing by diffusion results in the spread of the Jurkat cytoplasm after first cell fusion (2-2a). In 2-2a, the upper right cell of the Jurkat cell is unfused (pearl-chained) Jurkat cell that was attracted by DEP. The spread of the labeled iPS cytoplasm after first cell fusion (2-2b). Position of the nucleus of sacrificial iPS cell stained with Hoechst 33342 (2-2c). Scale bars show 20 *μ*m.

**FIG. 4.**
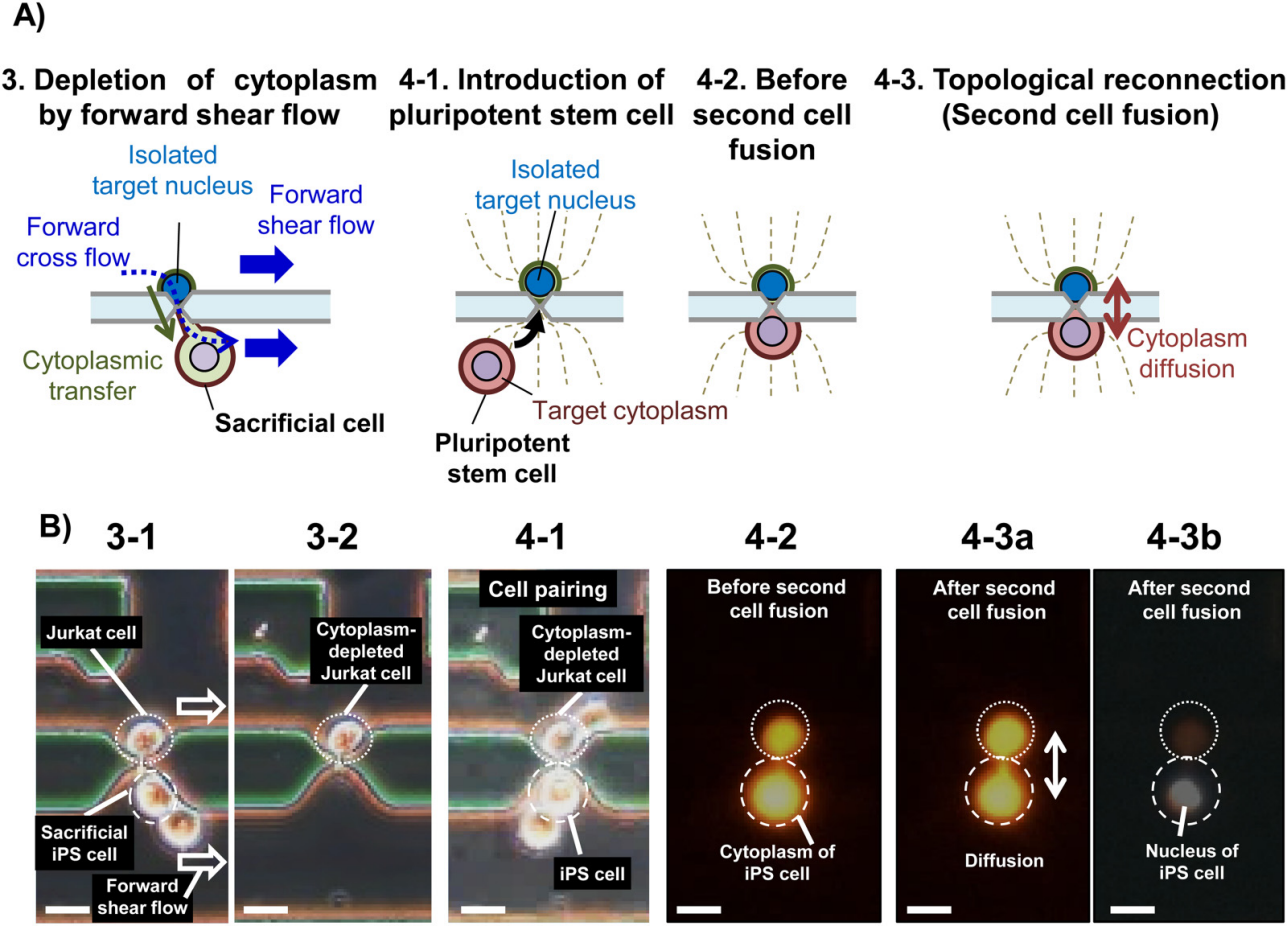
Topological reconnection involving shear flow-induced surgical removal of the sacrificial cell and subsequent fusion with a second pluripotent stem cell. (a) Schematics illustrating procedures for topological reconnection, including the generation of a forward shear flow to withdraw the cytoplasm of the somatic cell while removing the sacrificial cell (3). A pluripotent stem cell (4-1) is introduced and paired with the cytoplasm-depleted target cell (4-2) for the second cell fusion (4-3). (b) Actual images of topological reconnection showing the removal of sacrificial iPS cell to withdraw the cytoplasm of Jurkat cell and isolate the nucleus of Jurkat cell by forward shear flow (3-1, also video S2 in the supplementary material). The cytoplasm-depleted Jurkat cell remain trapped at the microslit by DEP (3-2). Introduction of a fresh iPS cell (second iPS cell) stained with Calcein Red-Orange to form a cell pair with the cytoplasm-depleted Jurkat cell via the microslit (4-1). Fluorescence image of a pair of second iPS cell and the cytoplasm-depleted Jurkat cell (4-2). The cytoplasm-depleted Jurkat cell in (4-2) also shows a Calcein Red-Orange fluorescence acquired during the first cell fusion with Calcein Red-Orange-stained sacrificial iPS cell [see also Fig. 3b(2-2b)]. Cytoplasm diffusion after the second cell fusion (4-3a). The last image (4-3b) shows the nucleus of iPS cell stained with Hoechst 33342. Scale bars show 20 *μ*m.

**FIG. 5.**
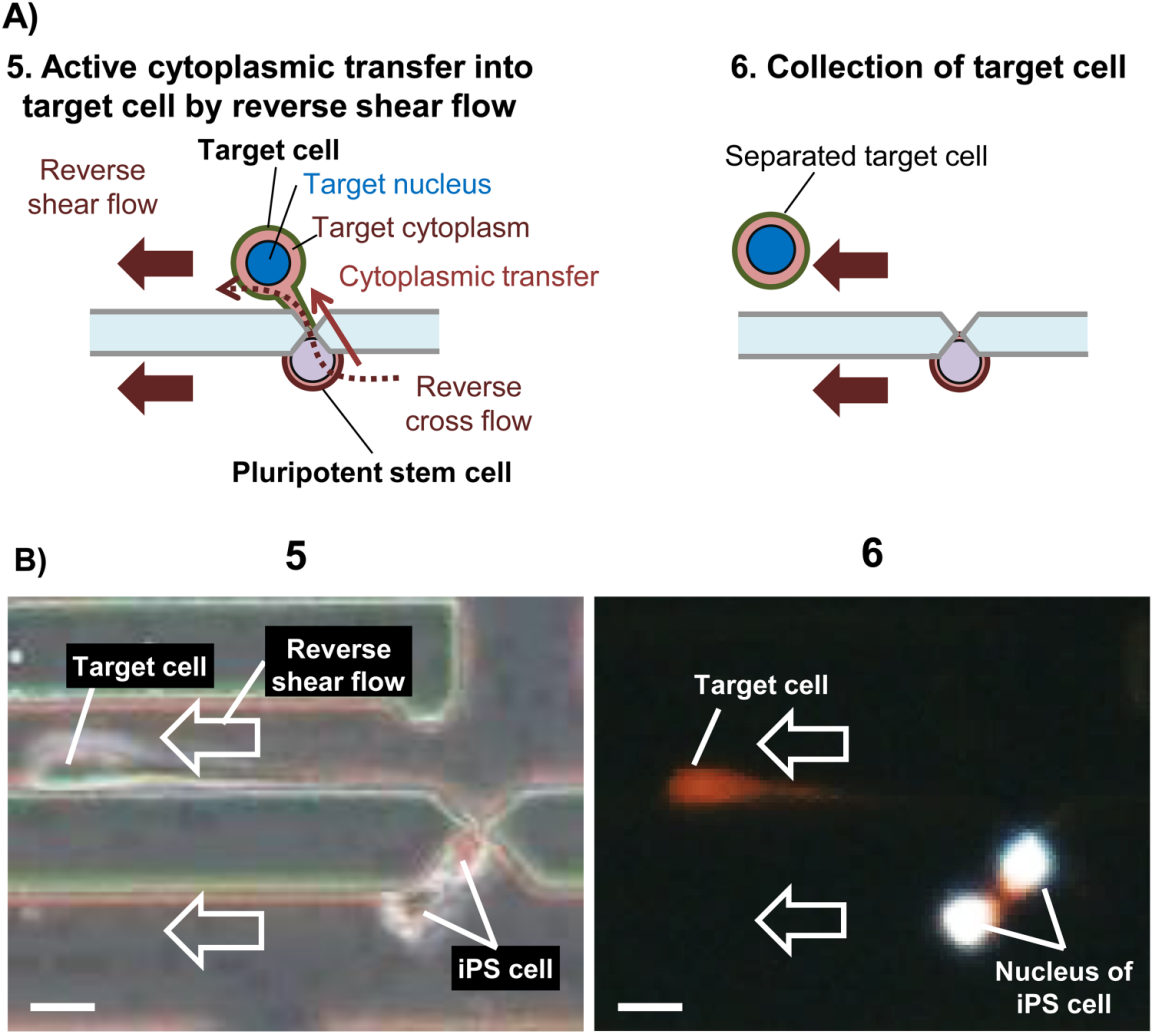
Procedures for harvesting target cells after cytoplasm transfer. (a) Illustrations of flow operations to actively transfer stem cell cytoplasm into a target cell by reverse shear flow (5). The target cell is pulled away and driven to a collection port by a contiguous-reverse shear flow generated in the collection channel (6). (b) Actual results showing the target cell being harvested by a reverse shear flow (video S3 in the supplementary material is another version). Harvesting of the target cell by application of a contiguous-reverse shear flow to drive it into the collection port (5). The position of the nucleus of an iPS cell (stained with Hoechst 33342) during the harvesting process is also shown (6). Scale bars show 20 *μ*m.

**FIG. 6.**
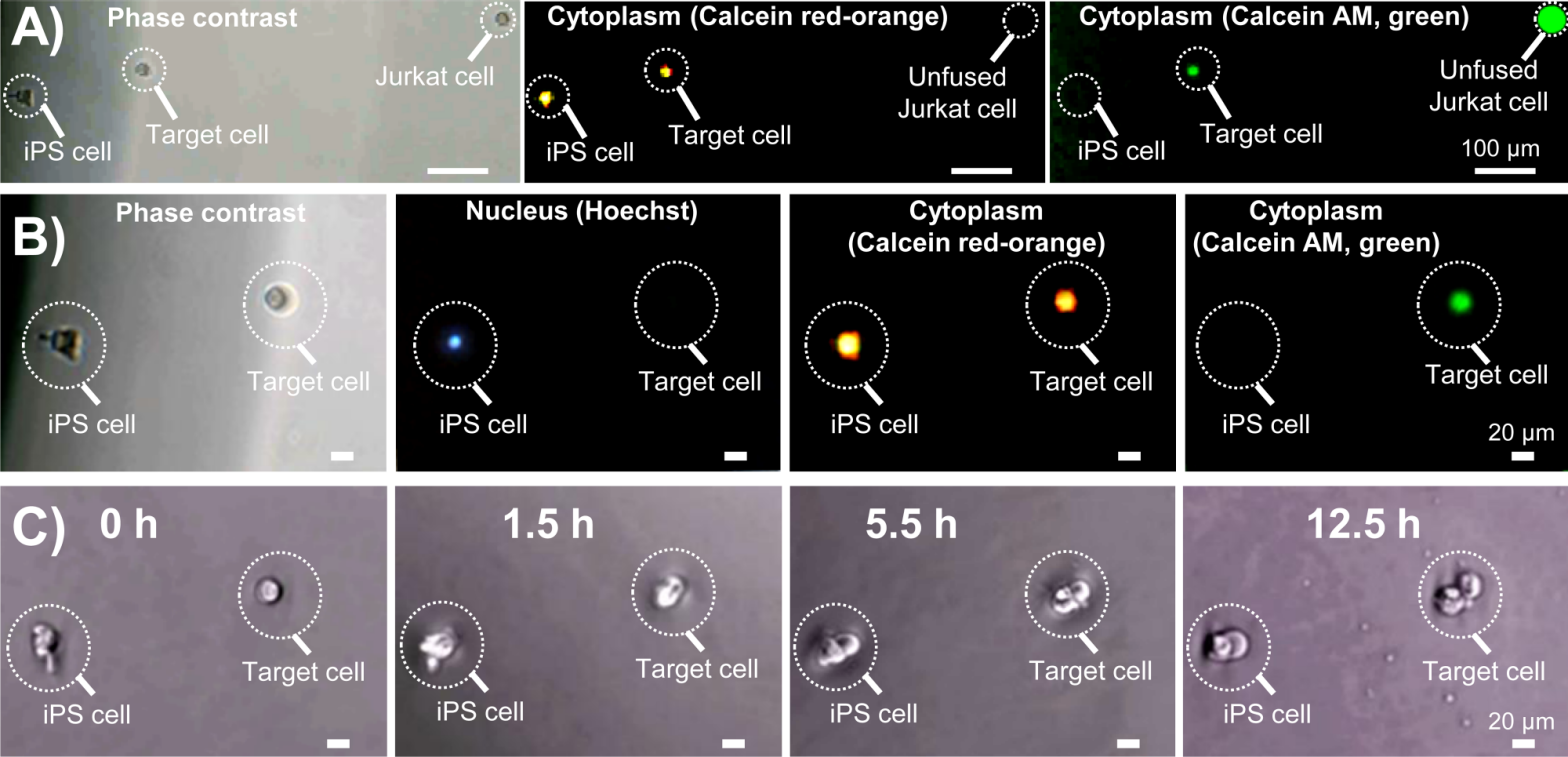
Culture and imaging of the harvested target cell. (a) Phase contrast and fluorescence images of a target cell, unfused iPS cell, and unfused Jurkat cell. Note that the harvested target cell contains the cytoplasm (Calcein Red-Orange) of an iPS cell as well as its own cytoplasm (Calcein AM). (b) Enlarged phase contrast and fluorescence images of a target cell and unfused iPS cell. Note that the unfused iPS cell showed the fluorescence (Hoechst 33342) of the iPS cell nucleus. (c) Imaging results showing cell division of a target cell cultured in a 96-well plate for 12.5 h.

## II. MATERIALS AND METHODS

### A. Cell culture and electrofusion reagents

As a proof of the concept to confirm the capability of the cell–cell topological reconnection technique, we opted to use Jurkat cells as model somatic cells since they are readily available and easy to culture compared with primary cell lines. Jurkat cell (Clone E6-1, ATCC® TIB-152^™^) was purchased from American Type Culture Collection (VA) and cultured in Dulbecco's Modified Eagle Medium (DMEM) supplemented with 10% FBS (fetal bovine serum) (both purchased from Thermo Fisher Scientific Inc., MA) under standard culture conditions (5% CO_2_ and 95% humidified air at 37 °C). For pluripotent stem cells, we used human iPS cells established from TIG1-4f lung fibroblasts that were provided by Professor Takashi Tada of Kyoto University, Japan.^20^ These cells were expanded and maintained on polystyrene culture dishes coated with 0.5 *μ*g/cm^2^ iMatrix (Wako Pure Chemical Industries, Ltd., Osaka, Japan), using a feeder-free StemFit® AK 02 N medium (Takara Bio Inc., Shiga, Japan) including 5 *μ*M Y-27632, MF (The Drug Master File System) (Wako Pure Chemical Industries, Ltd., Osaka, Japan), under standard culture conditions.

For visualization of cell fusion, the cytoplasm of the Jurkat cells was labeled with 1 *μ*g/ml Calcein AM (acetoxymethyl ester group) and that of the iPS cells with 1 *μ*g/ml CellTrace^™^ Calcein Red-Orange, AM (Calcein Red-Orange) for 5 min at 37 °C. The nuclei of the iPS cells were also labeled with 1 *μ*g/ml Hoechst 33342 for 5 min at 37 °C to trace the position of the nucleus in a fused cell. These fluorescence reagents were purchased from Thermo Fisher Scientific Inc., MA. Cell suspension including labeling the reagents was centrifuged at 600 rpm or 1000 rpm for 2 min and the pellets were resuspended in a low-conductivity cell fusion buffer. The cell fusion buffer consisted of 300 mM sorbitol, 0.1 mM calcium acetate, 0.5 mM magnesium acetate (purchased from Wako Pure Chemical Industries, Ltd., Osaka, Japan), and 1 mg/ml BSA (bovine serum albumin) (Sigma-Aldrich Co. LLC., Darmstadt, Germany). The electroconductivity of the cell fusion buffer was approximately 100 *μ*S/cm.

### B. Device fabrication

Polydimethylsiloxane (PDMS) microfluidic chip fabrication involved mold preparation and PDMS microfluidic chip casting.

Mold fabrication was performed according to a manual provided by the manufacturer of the resist used (Microchem Corp., MA). Briefly, negative photoresist, SU8 3025, was coated on a silicon wafer to a thickness of 25–45 *μ*m by spin coating. After soft baking (65 °C for 5 min and 95 °C for 15 min), the photoresist coated on the silicon wafer, it was exposed under a photomask that drew flow channels having a microslit (>3 *μ*m) with to ultraviolet light at approximately 21 mW power for 10 s. The fabrication of the SU8 mold was completed after postexposure baking (65 °C for 1 min and then 95 °C for 3 min) and development in a developer solution and isopropanol for 2 min.

The microfluidic chip was fabricated in house from PDMS (SILPOT 184, Dow Corning Toray Co., Ltd., Tokyo, Japan) using standard soft lithography. For PDMS microfluidic chip casting, a PDMS solution was prepared by mixing 1 g of the curing agent with 10 g of the prepolymer. The mixture was degassed for 1 h in a desiccator. Spacers with a thickness of 1 mm were placed on the sides of a mold to ensure that the thickness of the cured PDMS microfluidic chip would be 1 mm. The PDMS solution was poured into the mold and then covered with a microscope slide glass. The PDMS layer was cured into a PDMS microfluidic chip by heating it at 150 °C for 30 min, after which the microfluidic chip was released from the mold.

Two aluminum electrodes were patterned on a polystyrene culture dish (diameter: 60 mm) by standard physical vapor deposition and had a spacing of 400 *μ*m. The microfluidic chip was bonded onto the dish and aligned such that the microslit was positioned between the aluminum electrodes.

### C. Microfluidic device configuration and flow operation

Figure 2(a) shows a schematic of the device configuration for one-to-one electrofusion with electric field concentration used in this study. The device consists of a PDMS microfluidic chip, two electrodes, and a function generator via an amplifier connecting the electrodes [Fig. 2(b)]. It consisted of two loading ports for feeding the Jurkat cell and iPS cells, respectively, two main flow channels connecting the loading ports, a pressure chamber connecting the terminals of the two main flow channels, a one-side outlet connecting a syringe pump for controlling the transferring of cytoplasm and separation of fused cells, an insulator wall separating two main flow channels and having a microslit smaller than the cell diameters, a collection port for collecting harvested target cells, and a collection channel on the main flow channel 1 side connecting to the collection port [Fig. 2(c)]. DEP is generated via a microslit through an insulator wall between the electrodes by applying alternating current (AC: 1.0 MHz, 10 Vp-p) from an amplifier and a function generator. To initiate one-to-one electrofusion via the microslit, pulsation is also generated by applying current (100 *μ*s, 10 Vp). The channels were coated with 1.0 mg/ml bovine serum albumin solution to prevent cell adhesion during loading and subsequent operations. Then, a somatic cell and a sacrificial iPS cell are loaded and aligned by DEP to form a pair at the microslit [Fig. 2(d)].

As shown in Fig. 2(a), a syringe pump situated biasedly on the side of channel 2 side was used to generate two types of shear flow: forward shear flow and reverse shear flow for the purposes of cell loading, shear flow separation, and target cell harvesting.

Forward shear flow from the loading ports through the two main flow channels side to the pressure chamber side was generated by aspiration from the pump [Fig. 2(a), solid blue arrows]. Simultaneously, a forward cross flow [Fig. 2(a), dotted blue arrow] passing thorough the microslit from the main flow channel 1 toward the main flow channel 2 was generated.

Reverse shear flow was generated by discharging from the pump [Fig. 2(a), solid red arrows]. Contrary to the forward flow, this flow originated from the pressure chamber and flowed through the two main flow channels toward the loading ports, in the process generating a reverse cross flow [Fig. 2(a), dotted red arrow] emanating from the main flow channel 2 through the microslit toward the main channel 1.

Simultaneously, the reverse shear flow generated a contiguous shear flow in the collection channel [see Fig. 2(a)], which was used to further separate the target cell from the pluripotent stem cell and drive it into the collection port. The target cell was then manually collected using a pipette and transferred into a 96-well plate coated in advance with 0.5 *μ*g/cm^2^ iMatrix (dissolved in 0.2 ml of DMEM) for culture and observation. The target cell in the 96-well plate was identified by observing the green fluorescence signals of Calcein AM that was used to label the cytoplasm of Jurkat cells, the red fluorescence signals of Calcein Red-Orange that was used to label the cytoplasm of iPS cells, and the blue fluorescence signal of Hoechst 33342 that was used to label the nucleus of iPS cells. For time-lapse imaging, the target cells were cultured in DMEM at 37 °C and under 5% CO_2_ in a chamber located on the microscope stage.

## III. EXPERIMENTAL RESULTS

### A. Topological connection by one-to-one electrofusion via a microslit

Figure 3 and video S1 in the supplementary material show the results of first cell fusion (topological connection) between a Jurkat cell (model somatic cell) and a sacrificial iPS cell. Figure 3b(1) shows a Jurkat cell (main flow channel 1) and a corresponding sacrificial iPS cell (main flow channel 2) aligned by DEP at a microslit on the wall separating the two flow channels. To monitor cytoplasmic mixing, the cytoplasm of the Jurkat cell was labeled with Calcein AM [Fig. 3b(2-1a)], while that of the sacrificial iPS cell was labeled with Calcein Red-Orange [Fig. 3b(2-1b)]. Upon application of a pulse voltage, one-to-one electrofusion was initiated between the Jurkat cell and the sacrificial iPS cell via the microslit, generating the first fusion pair. Consequently, Calcein AM from the Jurkat cell and Calcein Red-Orange from the sacrificial iPS cell, respectively, diffused concomitantly between the fused cell pair, indicating topological connection and mixing of the two cytoplasm [Figs. 3b(2-2a) and 3b(2-2b)]. It should however be noted that the nucleus of the sacrificial iPS cell labeled with Hoechst 33342 remained separated since it could not pass through the narrow microslit even after cell fusion [Fig. 3b(2-2c)]. Thus, with this fusion technique, only cells in direct contact at the microslit were involved in fusion, making it possible to selectively exchange cytoplasmic contents between fused single cells without nuclei mixing.

### B. Shear flow-mediated single cell surgery for topological reconnection

Next, to selectively remove the sacrificial iPS cell from the Jurkat cell after topological connection, a forward shear flow was generated in the two main flow channels by aspirating from the pressure chamber. As the flow pulled away the sacrificial iPS cell, it carried with it the cytoplasm of the Jurkat cell, as illustrated schematically in Fig. 4a(3), and experimentally in Fig. 4b(3-1) and video S1 in the supplementary material. This process was assisted by a forward cross flow passing through the microslit from the main flow channel 1 to 2 [Fig. 4a(3)] and resulted in the isolation of the sacrificial iPS cell, while leaving behind a cytoplasm-depleted Jurkat cell that remained immobilized at the microslit by DEP [Fig. 4b(3-2)]. It should be noted that it is important to deplete somatic cytoplasm in somatic cells prior to fusion with iPS cells in the second fusion step (topological reconnection) so as to ensure that the iPS cytoplasm acquired at this step becomes dominant. This creates an ideal pluripotent environment that can be beneficial to the reprogramming of the somatic nucleus by reprogramming factors acquired from the iPS cell.

Figure 4b(4-1) shows a cell pair (unfused) consisting of the cytoplasm-depleted Jurkat cell (which had lost most of its cytoplasm during the forward shear flow separation) and a newly introduced iPS cell containing the target cytoplasm. It should be noted that the cytoplasm of the Jurkat cell exhibits a Calcein Red-Orange [Fig. 4b(4-2)] which is evidence that it contains the cytoplasm of the sacrificial iPS cell acquired during the first cell fusion [Fig. 3b(2-2b)]. This also implies that the cell has an intact cell membrane necessary to retain the little remaining cytoplasm, which is important for survival. Now, application of a second pulse voltage initiated one-to-one electrofusion (topological reconnection) between the cytoplasm-depleted Jurkat cell and the second iPS cell via the microslit [Fig. 4b(4-3a)]. Following reconnection with the iPS cell, the cytoplasm-depleted Jurkat cell exhibited a slight increase in size, indicating that it regained the cytoplasm from the former [Fig. 4b(4-3a)]. Indeed, fusion could be confirmed by the diffusion of Calcein Red-Orange from the cytoplasm of the iPS cell to the cytoplasm-depleted Jurkat cell. In addition, it could be also confirmed by the diffusion of residual Calcein AM fluorescence dye from the cytoplasm-depleted Jurkat cell into iPS cell (video S2 in the supplementary material). Remarkably, the nucleus of the second iPS cell also remained separated from that of the Jurkat cell during this topological reconnection [Fig. 4b(4-3b)].

Taken together, shear flow-mediated single cell surgery in this technique enables both selective removal of the sacrificial cell and the depletion of the target cell cytoplasm simultaneously, thereby yielding a somatic nuclear for the topological reconnection step. It should be noted that shear flow-mediated cell–cell surgery (separation) is a daunting task. Indeed, in a previous study by our group, shear flow separation after fusion was attempted using a device with two outlets, each separately connected to a suction pump such that suction pressure could be applied to each main channel independently.^21^ However, in this case, shear flow separation could not be achieved without the use of F-actin inhibitors such as cytochalasin D to aid the process. In addition, Jurkat cells or iPS cells in the two flow channels would occasionally accumulate at the microslits due to a large pressure gradient at the microslits between the two main flow channels when suction is performed independently for each channel using two pumps.

The microfluidic device developed in this study could overcome the above challenges and achieve shear flow-mediated single cell surgery with remarkable success. This can be attributed to the unique modification we introduced to the device, namely, the shared pressure chamber which connects to a syringe pump located biasedly on one side of the 2 main channels [Fig. 2(a)]. This modification contributed greatly to the improved success of shear flow separation because it eliminated the pressure imbalance between the 2 main channels, thereby suppressing the development of a large pressure gradient between the two channels. This made it possible to perform postfusion shear flow separation more stably. Moreover, since the syringe pump for fluid suction and discharge was positioned biasedly on one side of the two main channels, we could controllably generate cross flow through the microslit to aid in shear flow separation, cytoplasmic transfer, and single-cell harvesting.

### C. Cytoplasm transplantation and target cell harvesting by shear flow reversal

To enhance cytoplasm transplantation from the iPS cell into the Jurkat cell (now fused together), the direction of shear flow was reversed (reverse shear flow) such that a reverse cross flow developed across the microslit from the main flow channel 2 toward channel 1 [Fig. 2(a)]. The reverse shear flow in the two main channels was also from the pressure chamber toward the loading ports. The two shear flows were generated to selectively pull the target cell (with target nucleus and target cytoplasm) away from the iPS cell after the topological reconnection [Fig. 5a(5)]. Figure 5(b) shows the target cell undergoing shear flow-induced extension. The fluorescence of Calcein Red-Orange dye shows that the target cell indeed acquired the fluorescently labeled cytoplasm of the second iPS cell as was being pulled and finally separated from the iPS cell. It is worth noting that the reverse cross shear flow plays an important role in driving cytoplasmic flow from the iPS cell (larger cell) to the cytoplasm-depleted target cell, as clearly visible from video 3 in the supplementary material. In the absence of external forces associated with the cross flow, cytoplasmic transfer from a larger cell to a smaller cell would not naturally occur, considering the relationship between the membrane tension and a cell's internal pressure.

Now, another important aspect of the reverse cross flow passing through the microslit from the main flow channel 2 to 1 is that it creates a flow in the collection channel connecting the microslits to the collection port. Thanks to this flow in the collection channel, the target cell could be driven successfully to the collection port [Fig. 5b(6)] for collection and for further culture or analysis. In fact, once in the collection port, the target cell could be picked up using a micropipette and transferred to a 96-well plate for single-cell culture and microscopic analysis.

Figure 6(a) shows the target cell, unfused Jurkat cell, and unfused iPS cell transferred to a 96-well plate from the collection port of the PDMS fusion chip after cell–cell topological reconnection. As opposed to the iPS cell, the target cell exhibited fluorescence signals of both Calcein Red-Orange and Calcein AM (green), which was used to label the cytoplasm of the iPS cell and Jurkat cell, respectively [Fig. 6(a), middle panel]. This result confirms that the target cell contained the cytoplasm of the iPS cell, and partially its own original cytoplasm (stained green), as evidenced by the tiny green spot in the far right panel of Fig. 6(a). As can be confirmed from Fig. 6(b), the target cell did not have the blue fluorescence signal of Hoechst 33342 that was used to label the nucleus of iPS cells, implying that electrofusion via a microslit successfully prevented nuclei mixing as expected.

It should be noted that the data reported here are for 154 target cells that were collected from 59 experiments (N = 59) consuming 59 chips. This gives a success rate of ∼2.6 cells/experiment. Since our test device had only one microslit, all processes from cell fusion to separation were done sequentially, and at most, 5–6 cells could be successfully processed per any given experiment. Thus, the success rate of cell–cell topological reconnection method (which includes two rounds of cell fusion and shear flow separation) was more than 60%. The success of cell recovery from the chip, which is essentially the number of harvested target cells successfully transferred from the collection port to a 96-well plate, was approximately 80%. Now, the same chip could be used for sequential processing of more than 2 cells, and it took approximately 10 min to produce one target cell.

The most critical failure mode was the occurrence of air-lock or microbubbles that would prevent shear flows in the microchannels, resulting in relatively strong cross shear flows (both reverse and forward) across the microslit. Strong cross shear flows were detrimental in that they would cause the hybrid cells to slip through the microslit, impeding cell–cell separation. In addition, strong cross flows would cause perturbation of contact creation (between a somatic cell and an iPS cell) via the microslit, leading to increased chances of fusion failure. Therefore, it was important to degas PDMS microfluidics chips and introduce carefully the buffer into the microchannels.

Next, to test cell viability, target cells were collected manually from the PDMS chip and cultured in a 96-well plate for microscopic imaging. Figure 6(c) shows time-lapse images of a target cell and an iPS cell collected and cultured in the same well. As can be appreciated from the images, the target cell manifested the first cell division within 12.5 h, similar to the unfused iPS cell [Fig. 6(c), right panel]. Now, a total of 96 target cells were successfully transferred to a microwell plate for culture. Out of these, 7 cells exhibited cell division within the first 2 days of the microwell culture, whereas 89 cells failed to manifest cell division completely, with the majority of them showing apoptosislike cell death. We suspect that damage due to shear stresses, effect of fusion buffer, and/or cell cycle mismatch between the two fused cells may contribute to the low cell survival and proliferation. In our future work, we will improve the culture condition by fine-tuning the culture medium to fit the target cell and also explore cell cycle synchronization as strategies to increase the survival and proliferation of target cells obtained by our novel technique.

Overall, these results demonstrate that topological reconnection technique could achieve successful transfer of iPS cell cytoplasm to the target Jurkat cell while preventing nuclei mixing, and they highlight the fact that target cells generated by this technique are potentially viable and can undergo cell division under appropriate culture conditions.

## IV. DISCUSSIONS

This study has presented a novel technique, namely, the “cell–cell topological reconnection technique,” and demonstrated its application toward accomplishing transfer and insertion of a somatic cell nuclear into the cytoplasm of the pluripotent cell (Fig. 1). Integrating our original technology of one-to-one electrofusion with electric field concentration^12,21^ with shear flow-mediated single cell surgery, the topological reconnection technique was applied successfully to transplant iPS cell cytoplasm into a cytoplasm-depleted target somatic cell, generating a target cell that at the end contains an iPS cell cytoplasm and a somatic cell nucleus.

The cell–cell topological reconnection technique offers several advantages over conventional cell fusion methods. First, as stated already, it enables both fusion and cytoplasmic transplantation while preventing the mixing of nuclear materials, which is highly important where the introduction of exogenous genetic materials from a fusion partner has to be avoided to prevent polyploidy. Moreover, compared with traditional nuclear transfer techniques that rely heavily upon manual operation with microtools, the cell–cell topological reconnection technique allows for the exchange of the cytoplasm between different cell types (e.g., iPS cells and somatic cells) in a microfluidic channel, simply by fusion and flow control. Theoretically, cytoplasmic flow after topological connection would be from a smaller to a larger cell under the condition of no external force, considering that intracellular pressure is balanced by membrane tension, which is a function of curvature. However, the presence of cross flows in our topological reconnection technique implies the presence of an external force, which would contribute to the directional flow of the cytoplasm. Thus, under the influence of the cross shear flows, which are driven by pressure gradients between the main channels, cytoplasmic contents of an iPS cell (larger cell) would migrate to the cytoplasm-depleted target cell, as clearly presented in video 3 in the supplementary material. In other words, by reversing the flow in the main channels to reverse the direction of the cross flows, we could easily alter the direction of cytoplasmic flow between the fused cells, regardless of size differences.

Since conventionally used cell fusion approaches often yield tetraploid fusants,^25–28^ enucleation of oocytes is typically performed manually prior to nuclear transfer by electrofusion^29^ to avoid the formation of tetraploid fusants. However, this process of extracting the nuclear manually often involve cell lysis and/or membrane disruption to release the target nuclei.^30–32^ Moreover, it is practically difficult to exchange the nucleus of somatic cells or pluripotent stem cells using conventional microinjection pipettes, partly because, unlike oocytes which are large (>100 *μ*m in diameter), the diameter of these cells is considerably small (>10 *μ*m).^24^ In comparison, the cell–cell topological reconnection technique demonstrated in this study achieves cytoplasmic transplantation without cell lysis and with little disruption of the nuclear membrane, which can be beneficial to the survival of the derived target cells because damage to the cell membrane which often occurs during manual nuclear transfer operations can be avoided. Indeed, as shown in Fig. 6(c), the target cells exhibited division; dynamics which illustrate that the target cell was still viable. This is consistent with the results of previous studies by our group which showed that fusants obtained by one-to-one electrofusion were viable enough to produce daughter cells under standard culture conditions.^15,19^ Furthermore, fusion via microslits eliminates cell-size dependency, which is a major limitation of conventional techniques.

Since it accomplishes cytoplasmic transfer from a stem cell to a somatic cell through a straightforward two-step fusion and fluidic operations, this technique harbors potential applications in reprogramming studies. Indeed, previous studies have established that the cytoplasm of enucleated oocytes is capable of initiating cell reprogramming independently of the genetic materials in the nucleus. For example, it has been shown that nuclei extracted from fully differentiated somatic cells and inserted into the cytoplasm of enucleated oocytes can be fully reprogrammed.^22,23^ However, for regenerative medicine and stem cell therapy, nonintegrated autologous pluripotent stem cells generated from a patient's cell would be more desirable. Indeed, eliminating the use of viruses or vectors in somatic cell reprogramming remains the ultimate goal of reprogramming research. To this end, a more plausible approach would be to directly insert a somatic cell nucleus into the cytoplasm of an enucleated pluripotent stem cell like an ES/iPS cell to induce reprogramming by the acquired stem cell cytoplasm, as demonstrated in this study.

**FIG. 7.**
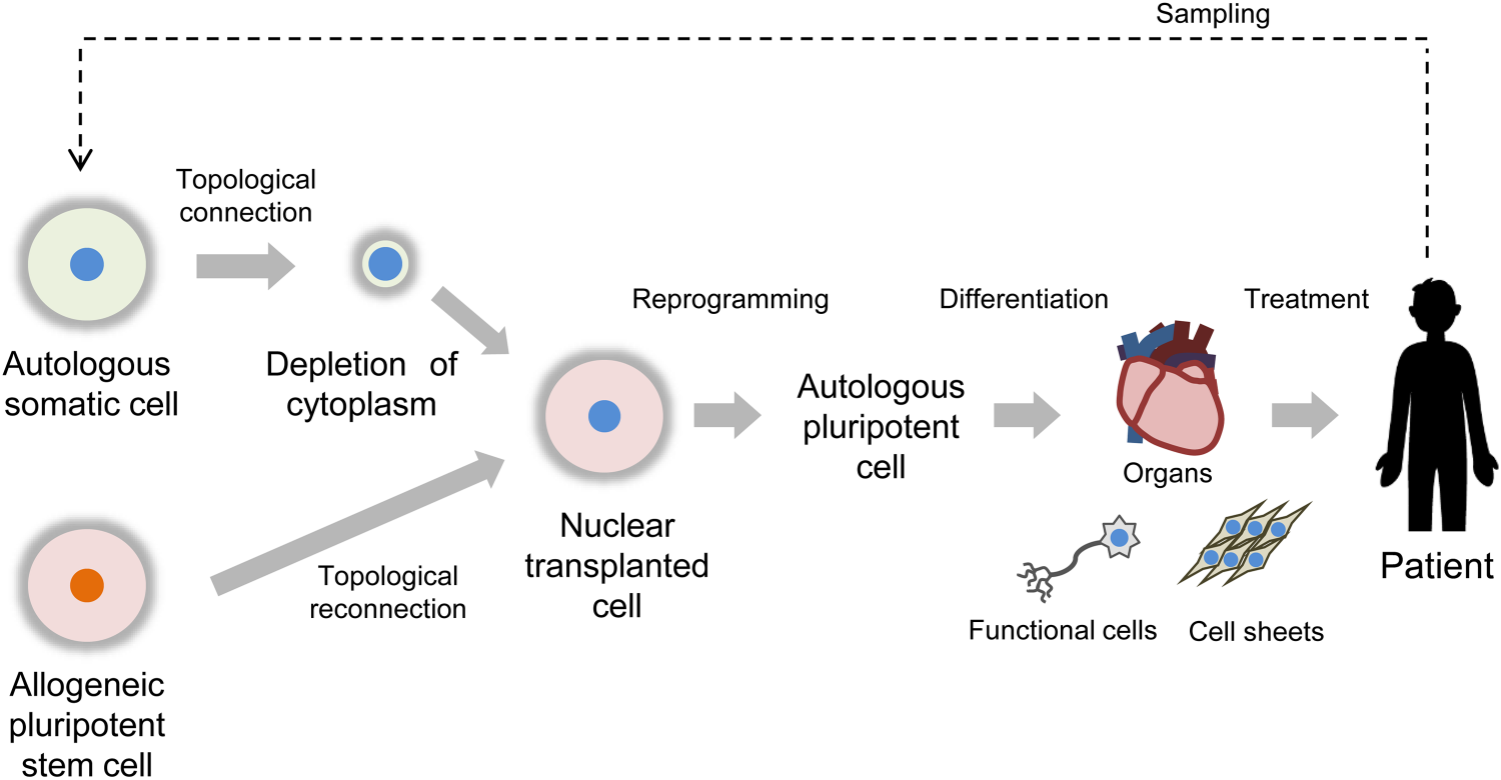
Concept of application of nuclear transplanted cells toward the generation of autologous pluripotent cell for regenerative medicine. An approach is proposed in which the nucleus of a somatic cell obtained from a patient's cell sample is transplanted into the cytoplasm of a pluripotent stem cell. Under a suitable culture condition, reprogramming of the nuclear transplanted cell can be triggered by pluripotency factors present in the cytoplasm of a stem cell, yielding a nonintegrated autologous pluripotent stem cell for stem cell therapy and generative medicine. The autologous stem cells can be differentiated into functional cells, cell sheets, or organs with which the patient is treated.

As outlined our concept in Fig. 7, we envision a scenario where, for instance, a somatic cell from a patient is depleted of its cytoplasm using the topological connection and then fed with the cytoplasm of a pluripotent stem cell by the topological reconnection to trigger its reprogramming by factors present in the cytoplasm of the pluripotent stem cell under suitable culture conditions. In this way, nonintegrated autologous pluripotent stem cells can be generated from a patient's cell for regenerative medicine. In this regard, the cell–cell topological reconnection technique based on one-to-one electrofusion with electric field concentration via microslits is a unique technique with potential applications in generating pluripotent cells for regenerative medicine. Importantly, the capability of selectively transferring the cytoplasm of an iPS cell into a target somatic cell while excluding its nucleus may open the possibility of generating autologous iPS cells whereby the cytoplasm of a pluripotent stem cell is used to reprogram the nucleus of a somatic cell.

To improve further the applicability of the technique, we aim to develop a device with higher throughput and yield in our future work. This will be achieved by taking advantage of the microfluidic platform to introduce automation and also develop a chip platform that allows for simultaneous fusion of arrayed single cell pairs. For example, arrayed microslits can be fabricated to obtain multiple fusants, and then use the same flow operations to perform cytoplasmic transplantation between the arrayed pairs simultaneously. Indeed, our group has previously demonstrated that one-to-one electrofusion with multiple orifices is possible.^16^ We have also demonstrated that it is possible to harvest a single cell after the cytoplasm exchange operations and transfer it to a 96-well plate for culture and microscopic observation. Indeed, we have shown that the cell harvested after the cytoplasm exchange could remain intact and viable [Fig. 6(c)]. Although the transfer to a well was done manually with a micropipette, the chip can be improved further to allow for automated cell collection and downstream single-cell analysis by connecting the fusion chip directly to a 96-well plate.

## V. CONCLUSION

In this study, we developed a cell–cell topological reconnection technique based on one-to-one electrofusion with electric field concentration to transplant the cytoplasm of a somatic cell into the nucleus of a pluripotent cell. To prevent nuclei mixing during cytoplasmic exchange between the fused cell pair while still permitting topological reconnection, one-to-one electrofusion of a Jurkat cell and an iPS cell was conducted successfully via a microslit whose width was smaller (∼4 *μ*m) than the nuclei. Then, using microfluidic operations involving two-step fusion and shear flow mediated single cell surgery, the cytoplasm of a somatic cell (a Jurkat cell with target nucleus) was removed and replaced with target cytoplasm from an iPS cell. The resultant target cell had the target nucleus of the Jurkat cell and the target cytoplasm of the iPS cell but not the nucleus of the iPS cell. Thus, cell–cell topological reconnection technique is a powerful tool for the processing involved in transformation of a target nucleus by foreign factors contained in target cytoplasm, opening the possibility of generating autologous pluripotent stem cell for applications to regenerative medicine in the future.

## SUPPLEMENTARY MATERIAL

See the supplementary material for three videos that have been provided to aid in the understanding of the study results. Video S1 shows cell loading, DEP alignment, and topological connection during the first electrofusion via microslit inside a PDMS microfluidic chip. Video S2 shows topological reconnection involving shear flow-induced surgical removal of the sacrificial cell and subsequent fusion with a second pluripotent stem cell. Finally, video S3 shows harvesting target cells after cytoplasm transfer.
